# Epigenetic Profiling of H3K4Me3 Reveals Herbal Medicine Jinfukang-Induced Epigenetic Alteration Is Involved in Anti-Lung Cancer Activity

**DOI:** 10.1155/2016/7276161

**Published:** 2016-03-20

**Authors:** Jun Lu, Xiaoli Zhang, Tingting Shen, Chao Ma, Jun Wu, Hualei Kong, Jing Tian, Zhifeng Shao, Xiaodong Zhao, Ling Xu

**Affiliations:** ^1^Shanghai Center for Systems Biomedicine, School of Biomedical Engineering, State Key Laboratory on Oncogene and Bio-ID Center, Shanghai Jiao Tong University, 800 Dongchuan Road, Shanghai 200240, China; ^2^Tumor Institute of Traditional Chinese Medicine, Longhua Hospital, Shanghai University of Traditional Chinese Medicine, 725 South Wanping Road, Shanghai 200032, China; ^3^College of Life Science, Northwest University, 229 Taibai Road, Xi'an 710069, China; ^4^Department of Oncology, Yueyang Hospital of Integrated Traditional Chinese and Western Medicine, Shanghai University of Traditional Chinese Medicine, 110 Ganhe Road, Shanghai 200437, China

## Abstract

Traditional Chinese medicine Jinfukang (JFK) has been clinically used for treating lung cancer. To examine whether epigenetic modifications are involved in its anticancer activity, we performed a global profiling analysis of H3K4Me3, an epigenomic marker associated with active gene expression, in JFK-treated lung cancer cells. We identified 11,670 genes with significantly altered status of H3K4Me3 modification following JFK treatment (*P* < 0.05). Gene Ontology analysis indicates that these genes are involved in tumor-related pathways, including pathway in cancer, basal cell carcinoma, apoptosis, induction of programmed cell death, regulation of transcription (DNA-templated), intracellular signal transduction, and regulation of peptidase activity. In particular, we found that the levels of H3K4Me3 at the promoters of* SUSD2, CCND2, BCL2A1,* and* TMEM158* are significantly altered in A549, NCI-H1975, NCI-H1650, and NCI-H2228 cells, when treated with JFK. Collectively, these findings provide the first evidence that the anticancer activity of JFK involves modulation of histone modification at many cancer-related gene loci.

## 1. Introduction

Chromatin is the macromolecular complex of DNA and histone proteins that provides the scaffold for packaging the eukaryotic genome [1, 2]. Histones H2A, H2B, H3, and H4 are the basic components of nucleosomes, which form the fundamental unit of chromatin [3, 4]. Chemical modifications to the histones alter chromatin structure and regulate gene expression by altering noncovalent interactions within and between nucleosomes [2, 5]. H3K4Me3 is an active histone modification which is positively associated with gene expression [3, 6]. Previous studies have shown that the levels of H3K4Me3 modification are closely associated with the development, treatment, and diagnosis of disease [7–9]. Chromatin immunoprecipitation followed by sequencing (ChIP-seq) has been developed to systematically characterize the contribution of epigenetic regulation in various biological processes via genome-wide profiling of various chemical modifications of histone proteins and genomic DNA methylation [10].

Lung cancer has become the leading cause of cancer-related deaths worldwide [11]. Overall, only 16.8% of patients with lung cancer survive five years after their first definite diagnosis, mainly as a consequence of uncontrollable cell proliferation or tumor metastasis [12, 13]. Although various therapeutic interventions, including surgery, chemotherapy, and radiotherapy, have been developed to prolong the survival time of patients, drug side effects, pain, and emaciation are still significant problems for tumor sufferers and their physicians [14, 15].

Traditional Chinese medicine (TCM) has been employed in the treatment of many diseases for several thousand years and still plays an important role in public health in present day [16]. Jinfukang (JFK), a TCM formula, has been clinically used for treating lung cancer patients for more than ten years, and its curative effects have been reported to be promising [14, 17–19]. However, although the clinical effects of JFK are known [17], its antitumor mechanism remains unclear. In previous study, researchers demonstrated that JFK modulates the expression of genes which are involved in the cell cycle and apoptosis [18]. In the present study, we examined the pattern with H3K4Me3 pattern modulated by JFK in A549 cells and the implication in its antitumor activity of JFK.

## 2. Materials and Methods

### 2.1. Animals and Treatments

All animals received humane care during the study, under a protocol that was in accordance with institutional guidelines. Sprague-Dawley rats were obtained from Shanghai SLAC Laboratory Animal Co., Ltd. All rats were fed laboratory rat-fodder and water, ad libitum, and were maintained at 22 ± 1°C and lighting was set for 12 h on, 12 h off. In this experiment, 20 rats were divided into two groups including control and JFK. Rats were administrated orally 1 mL saline for control group and 4 g Kg^−1^ body weight JFK (volume: 1 mL) for JFK group, respectively, three times a day for 15 consecutive days.

### 2.2. JFK Drug Serum Preparation

The procedures of drug serum preparation were performed according to a previous study [20]. Briefly, two hours after the last administration, all blood samples were collected by abdominal aortic method under the state of anesthesia using pentobarbital sodium. Each rat was sacrificed by cervical dislocation. The concretionary blood was centrifuged at 1,500 g for 15 min, and then the JFK drug serum was collected and stored at −80°C.

### 2.3. Cells and Cell Culture

The human lung cancer cell lines A549, NCI-H1975, NCI-H1650, and NCI-H2228 were obtained from the Shanghai Institute of Biochemistry and Cell Biology. Cells were cultured in RPMI-1640 Medium (Gibco, USA) and maintained at 37°C in a humidified atmosphere of 5% CO_2_. Growth media were supplemented with 10% fetal bovine serum (FBS) (Gibco, USA) and penicillin/streptomycin (Life Technologies, Inc., USA) final concentration is 100 U/mL. The cells were subcultured when they reached approximately 90% confluence using a 0.25% trypsin solution. For observation of JFK-induced cell alteration, 10% control serum and JFK drug serum were supplemented to growth media for replacing 10% FBS, respectively.

### 2.4. Cell Viability Analysis

Cells were cultured in 96-well plates overnight and incubated with indicated serums for 12 h, 24 h, 48 h, 72 h, 96 h, and 120 h, respectively. Cellular viability was measured by Cell Counting Kit 8 (CCK8) (Dojindo, Japan). In brief, the cell proliferation reagent WST-8 (10*μ*L) was added to each well and cells were incubated for 2 h at 37°C. Viable cell numbers were estimated by measuring the optical density (OD) at 450 nm. Absorbance of control group was set as 100% viability, and absorbance of cell-free wells containing medium was set as zero.

### 2.5. DAPI Staining

A549 cells were fixed in 4% paraformaldehyde and then stained with DAPI (Sigma, USA) and observed using Confocal Laser Scanning Microscopy (CLSM) (Nikon, Japan). Cells were viewed to be damaged if the dissolution, condensation, or fragmentation of nuclei was observed.

### 2.6. PI Staining

A549 cells were fixed, stained with PI (Sigma, USA), and observed using fluorescence microscopy (Nikon, Japan). Pictures were obtained through fluorescence microscopy at the excitation wavelength of 536 nm. Cells were designated as being dead if they had highly red color and nondead if the staining was slight or none.

### 2.7. Cell Apoptosis Analysis

Determination of phosphatidylserine (PS) and membrane integrity was performed by Annexin V-FITC/PI Apoptosis kit (Zoman, China). In brief, A549, NCI-H1975, NCI-H1650, and NCI-H2228 cells were harvested by trypsin (no EDTA) and washed twice with PBS, then stained with Annexin V-FITC/PI, and analyzed by flow cytometry (BD LSRFortessa, USA).

### 2.8. Chromatin Immunoprecipitation (ChIP) Assay

ChIP assay was performed as previously described [10]. In brief, 3.5 × 10^6^ cells were rinsed with PBS, cross-linked with 1% formaldehyde and quenched with 2.5 M glycine (Sigma, USA), and then harvested in a 1.5 mL tube. 100 bp to 300 bp chromatin fragments were obtained after sonication with indicated condition. 1% total fragmented DNA was transferred to a new tube set as input. Immunoprecipitated DNA fragments were captured by H3K4Me3 antibody (Abcom, USA), following quantification by Picogreen measurement kit. Primers of* MYC* and* EGFR* were designed for detecting whether the immunoprecipitated DNAs were enriched and qualified for library procedure. Primer sequences were showed in Table 1.

### 2.9. Library Preparation and ChIP-Seq

For library, 40~50 ng immunoprecipitated DNAs and input DNAs were end-repaired and ligated adapters to the DNA fragments using NEBNext Ultra End-Repair/dA-Tailing Module and NEBNext Ultra Ligation Module (NEB, USA), respectively. DNA size selection was performed using 2% Agarose Gel; then 200~500 bp DNA fragments were excised and purified using Qiagen Gel Extraction Kit (Qiagen, Germany). Each sample was amplified for 14 cycles in a DNA thermal cycler using Q5 High-Fidelity DNA Polymerase (NEB, USA) and corresponding PCR Master Mix. Lastly, the PCR products were quantified using Nanodrop and performed standard single end sequencing with 50 bp reads using Illumina Hiseq 2000 (Illumina, USA). The raw sequencing data of this study are available in the EMBL database under accession number E-MTAB-3992: http://www.ebi.ac.uk/arrayexpress/.

### 2.10. Bioinformatic Analysis

Raw data were extracted and initially analyzed by Illumina software. We used FastQC software to detect data quality. After prefiltering the raw data by removing sequenced adapters and low quality reads, the sequence tags were aligned to human genome (NCBI/hg19) with Bowtie alignment tool [21]. After being filtered, at least 12 million unique reads per sample were used for peak detection. Peak detection was performed using the MACS program with a two-sample analysis where sequenced input (1%) was used as negative control [22]. Due to the differences in the number of reads between experimental data and input DNA data, a random subselection was applied to the larger data set for normalization. The statistics of RNA-seq data sets were shown in Table 2.

For identifying qualified peak regions, experimental data and input DNA data were consolidated in a window size of 100 bp and *P* value < 0.05. To compare JFK-treated versus control H3K4Me3 ChIP data sets, the two lists of peaks were matched based on overlapped genome positions. Matched peak regions were annotated using UCSC annotation database and genes with qualified peaks within 2,000 bp of the Transcriptional Start Sites (TSSs) were identified. Fold change was also calculated for each matched peak region comparing JFK-treated versus control H3K4Me3 peaks.

The Database for Annotation, Visualization and Integrated Discovery (DAVID) bioinformatics resource was used to annotate gene functions and pathways [23]. Briefly, Gene Ontology (GO) analysis [24] and Kyoto Encyclopedia of Genes and Genomes (KEGG) pathways analysis [25] were performed by uploading differential gene list to DAVID Bioinformatics Resources 6.7. Besides, network construction was performed using Reduce Visualize Gene Ontology (http://revigo.irb.hr/), and then network graph was modified using Cytoscape software (version 3.2.1).

### 2.11. ChIP-qPCR Validation

Immunoprecipitated DNAs from control and JFK samples were obtained by ChIP assays which are described as previously mentioned. For validation of target genes H3K4Me3 modification fold change, all target gene primers were designed using Primer3 web (version 4.0.0). All primer sequences are showed in Table 1.

### 2.12. Quantification of mRNA Level

Transcription level of genes of interest was evaluated using quantitative real-time PCR (RT-qPCR) with SYBR Green PCR Master Mix (Bestar, Germany). RT-qPCR was performed according to standard procedure. All primer sequences used for RT-qPCR were shown in Table 1.

### 2.13. Statistical Analysis

Data are presented as the mean ± SD. The differences between the groups were examined using the standard one-way ANOVA. SPSS for Windows 14.0 software package was used. Differences were considered significant at *P* < 0.05.

## 3. Results

### 3.1. A549 Cell Viability and Cell Nucleus Morphology Are Changed after Incubation with JFK

**Figure 1:**
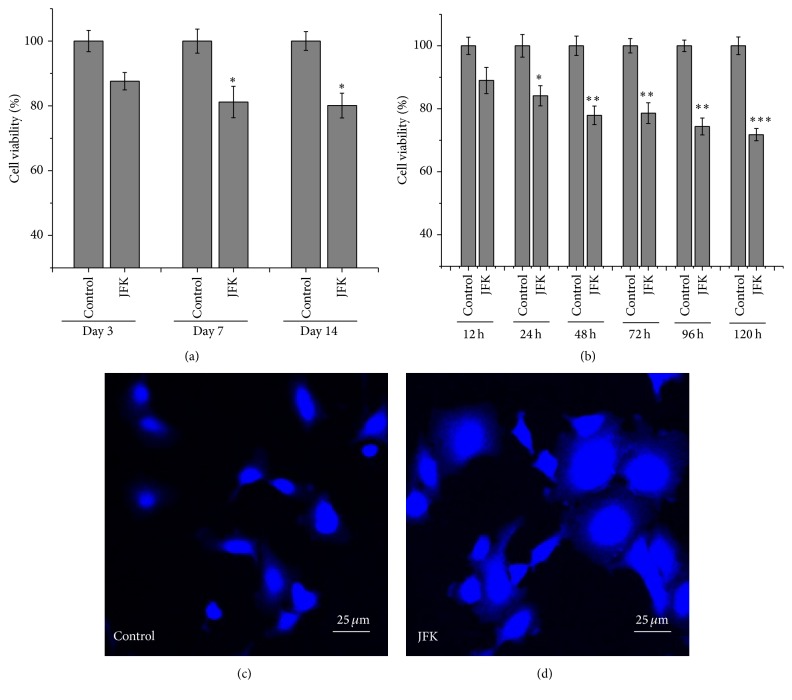
Treatment with JFK induces proliferation inhibition and nuclear morphology alteration in A549 cells. (a) A549 cells were treated with JFK drug serums from day 3, day 7, and day 14, respectively (*n* = 3, ^*∗*^ *P* < 0.05). (b) A549 cells were treated with JFK drug serum from day 14 for 12 h, 24 h, 48 h, 72 h, 96 h, and 120 h, respectively (*n* = 3, ^*∗*^ *P* < 0.05, ^*∗∗*^ *P* < 0.01, and ^*∗∗∗*^ *P* < 0.001). (c, d) A549 cells were stained with DAPI for detecting nucleus changes using CLSM. Scale bar: 25 *μ*m.

The survival rate and changes to the nuclear morphology of A549 cells were monitored via a multimode reader and optical microscopy. We observed that cell viability decreases significantly after 24 h incubation with JFK drug serums obtained from day 7 and day 14 (Figure 1(a)). In addition, time-dependent assays suggest that cell viability reduces significantly when A549 cells are exposed to the JFK serum from day 14 for incubation times longer than 24 h (Figure 1(b)). Thus, we chose a 48 h incubation time with JFK for all future experiments. Interestingly, nuclear staining with DAPI revealed alterations to the nuclear morphology in response to JFK. As shown in Figures 1(c) and 1(d), after the JFK treatment for 48 h, many nuclei were markedly larger compared to those in the control, indicating that these cells are in a state of abnormality.

### 3.2. Proapoptotic Effects of JFK on A549 Cells

**Figure 2:**
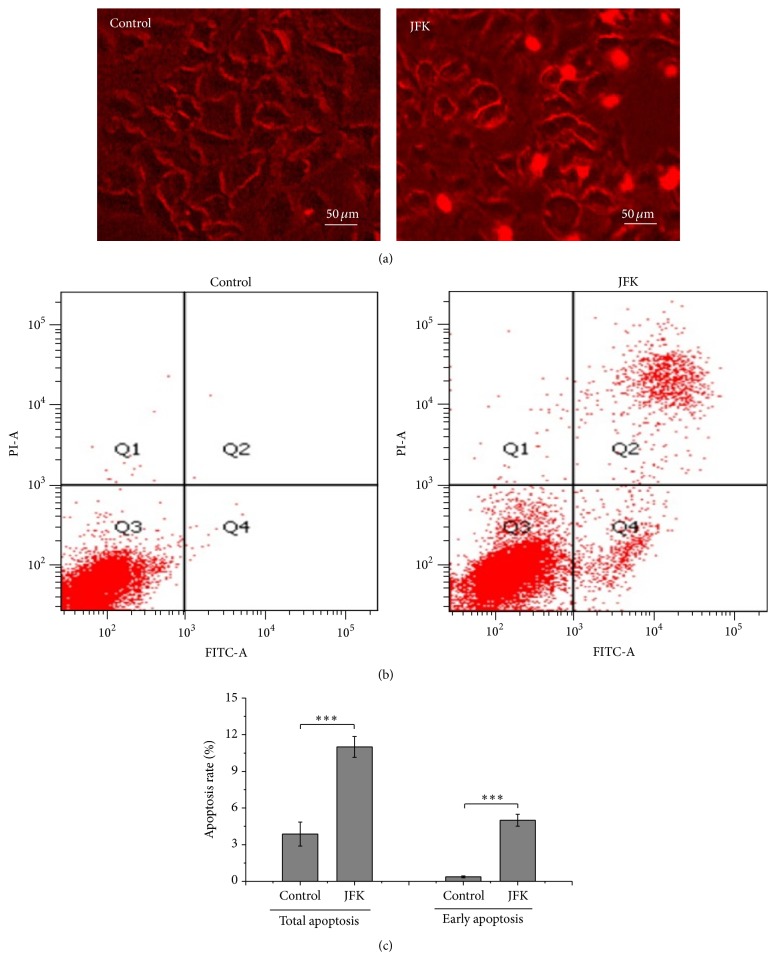
Treatment with JFK induces cell death and apoptosis in A549 cells. (a) A549 cells were stained with PI for detecting deathbed cells using fluorescence microscope. Scale bar: 50 *μ*m. (b) A549 cells were treated with JFK, and the apoptotic progression was determined by flow cytometry. (c) Total apoptosis and early apoptosis ratios were analyzed based on results of flow cytometry (*n* = 3, ^*∗∗∗*^ *P* < 0.001).

To further understand the effects of JFK on A549 cells, we examined the number of apoptotic cells by staining with PI and using fluorescence microscopy. As shown in Figure 2(a), cells colored red, indicative of apoptosis, are found following treatment with JFK, suggesting that JFK could induce apoptosis in these cells. Flow cytometric analysis also showed a significant increase in JFK-induced apoptosis compared to a vehicle treatment control (Figure 2(b)). In particular, the total apoptosis rate calculated as Annexin and PI positive cells and the early apoptosis rate calculated as Annexin positive cells, respectively, by flow cytometry were significantly increased in A549 cells after JFK treatment, compared to those in vehicle treated A549 cells (Figure 2(c)).

### 3.3. JFK Induces an Alteration in the H3K4Me3 Pattern in A549 Cells

**Figure 3:**
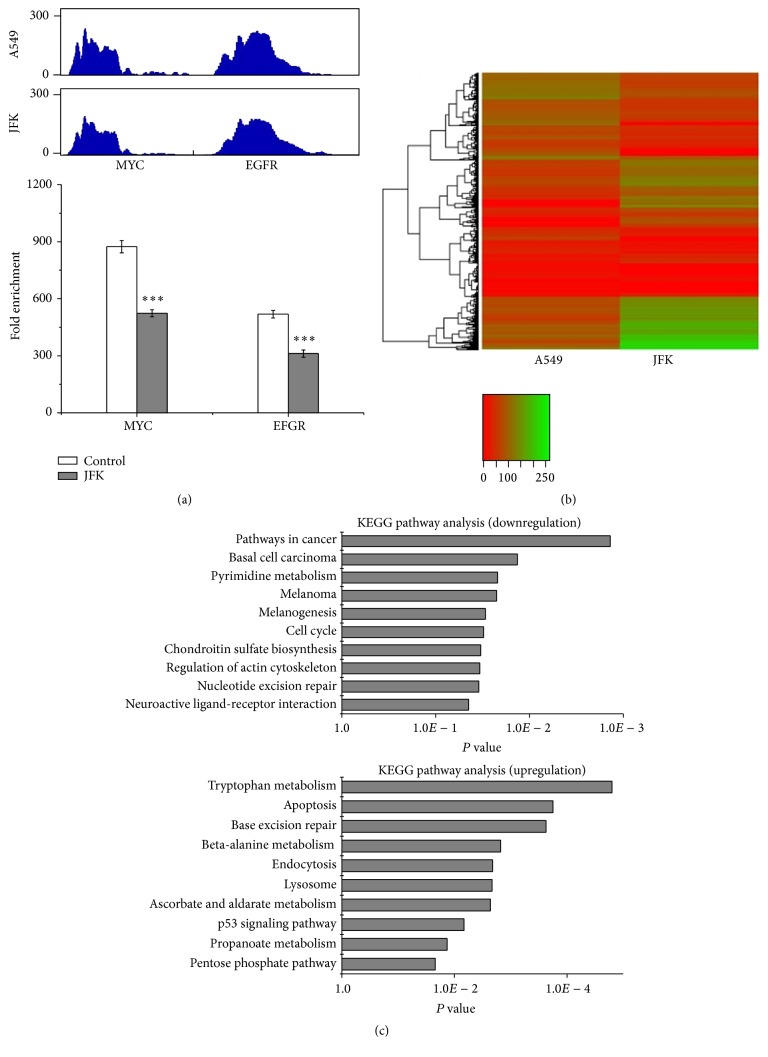
Gene Ontology analysis of JFK-induced A549 cell H3K4Me3 modification altered genes. (a) ChIP tag density profiles of H3K4Me3 modification at TSSs on* MYC* and* EGFR* genes. Fold change of* MYC* and* EGFR* H3K4Me3 modification status are detected by ChIP-qPCR (*n* = 4, ^*∗∗∗*^ *P* < 0.001). (b) Heat map of top 2000 H3K4Me3 modification altered genes from upregulation and downregulation, respectively, in A549 cells with or without JFK treatment (*P* < 0.05). (c) KEGG pathway analysis of upregulated and downregulated genes.

Previous studies have reported that the level of H3K4Me3 modification in TSSs is linked to transcriptional activation [3, 6]. To examine the alteration of H3K4Me3 modification upon exposing A549 cells to JFK, we performed genome-wide mapping of H3K4Me3 modification by ChIP-seq. Firstly, we examined* MYC* and* EGFR*, two genes which are known to have high levels of H3K4Me3 in A549 cell. As shown in Figure 3(a), the degree of H3K4Me3 at these genes significantly decreases following treatment with JFK. To further characterize these changes, a genome-wide peak detection analysis was performed using the MACS program. Compared to control, the H3K4Me3 enrichment status at many TSSs in the JFK-treated cells was significantly altered (Table S1 in Supplementary Material available online at http://dx.doi.org/10.1155/2016/7276161). A heat map analysis of the top 2,000 genes showing altered levels of H3K4Me3 (*P* < 0.05), including those exhibiting lower and greater levels of modification, identified promising candidates for further bioinformatic analysis (Figure 3(b)).

### 3.4. Gene Ontology Analysis of Genes with Altered H3K4Me3 Modification after JFK Treatment

We next analyzed the H3K4Me3 modification fold enrichment within 2,000 bp of TSSs between control- and JFK-treated A549 cells. We then identified the top 2,000 genes exhibiting altered levels of H3K4Me3 (*P* < 0.05) for further GO analysis and KEGG pathway analysis. The top ten GO terms are presented in Table 3. These results suggest that treatment with JFK could downregulate H3K4Me3 modification levels at those genes involved in many pathways including ion transport, Wnt receptor signaling pathway, and positive regulation of transferase activity. By contrast, those genes with greater levels of H3K4Me3 following JFK treatment are involved in programmed cell death, induction of apoptosis, positive regulation of T cell activation, and so forth.

**Figure 4:**
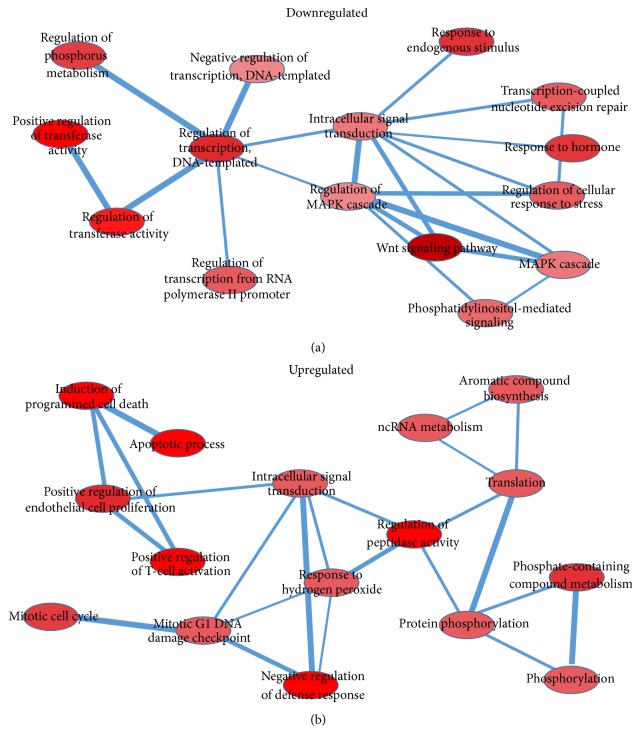
Network analysis of upregulated and downregulated genes. (a, b) Parts of top 50 GO terms from downregulated and upregulated genes, respectively, have mutual effect between each other. Degree of red color represents the *P* value of GO terms (the lower *P* value, the more red).

KEGG pathway analysis suggests that the downregulated genes are enriched in pathways involved in pathways in cancer, basal cell carcinoma, pyrimidine metabolism, and so forth, whereas the upregulated genes are enriched in those pathways involved in tryptophan, apoptosis, base excision repair, and so forth (Figure 3(c)). Network analysis of the top 50 downregulated GO terms in the JFK-treated A549 cells identified key signal transduction networks affected. As shown in Figure 4(a), three prominent GO terms associated with the downregulated genes include regulation of transcription (DNA-templated), intracellular signal transduction, and regulation of MAPK cascade. For top 50 upregulated GO terms, regulation of peptidase activity plays an important role in the whole network which can interact with induction of programmed cell death and apoptotic process indirectly (Figure 4(b)). Thus, overall, this Gene Ontology analysis suggests that JFK-induced apoptosis could be attributed to alteration in the levels of H3K4Me3 modification in many tumor-related genes.

### 3.5. JFK Induces H3K4Me3 Alteration in Specific Tumor-Related Genes in A549 Cells

**Figure 5:**
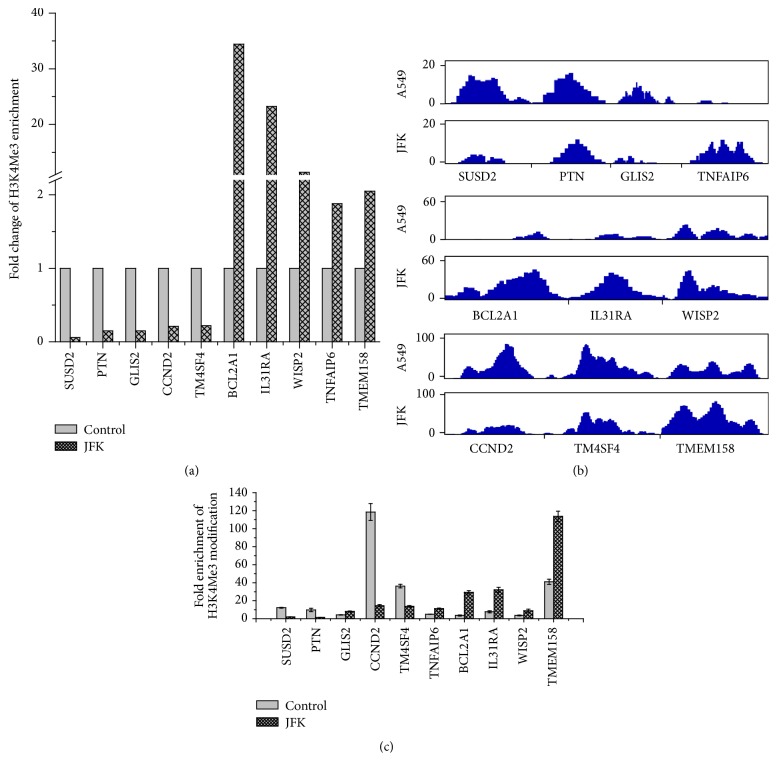
Analysis of H3K4Me3 pattern in the promoter regions of tumor-related genes after exposure to JFK. (a) Analysis of H3K4Me3 modification fold change in tumor-related genes based on ChIP-seq data. (b) ChIP tag density profiles of H3K4Me3 modification at TSSs on tumor-related genes. (c) Verification of H3K4Me3 modification altered genes using ChIP-qPCR (*n* = 3).

To further understand the relationship between JFK-induced effects on A549 cell and changes in H3K4Me3 levels at tumor-related genes, we analyzed the modification changes at five survival-regulated genes (*SUSD2, PTN, GLIS2, CCND2,* and* TM4SF4*) and five apoptosis-regulated genes (*BCL2A1, IL31RA, WISP2, TNFAIP6,* and* TMEM158*). As shown in Figure 5(a), treatment of A549 cells with JFK for 48 h downregulated the H3K4Me3 modification levels at* SUSD2, PTN, GLIS2, CCND2,* and* TM4SF4* genes, whereas those at* BCL2A1, IL31RA, WISP2, TNFAIP6,* and* TMEM158* genes were upregulated compared to those genes in control. The ChIP tag density profiles also matched these fold change results (Figure 5(b)). We also performed ChIP-qPCR to validate these high-throughput findings, and all genes, except* GLIS2*, showed similar changes (Figure 5(c)).

### 3.6. Verification of JFK-Induced H3K4Me3 Alteration in Other Lung Cancer Cell Lines

**Figure 6:**
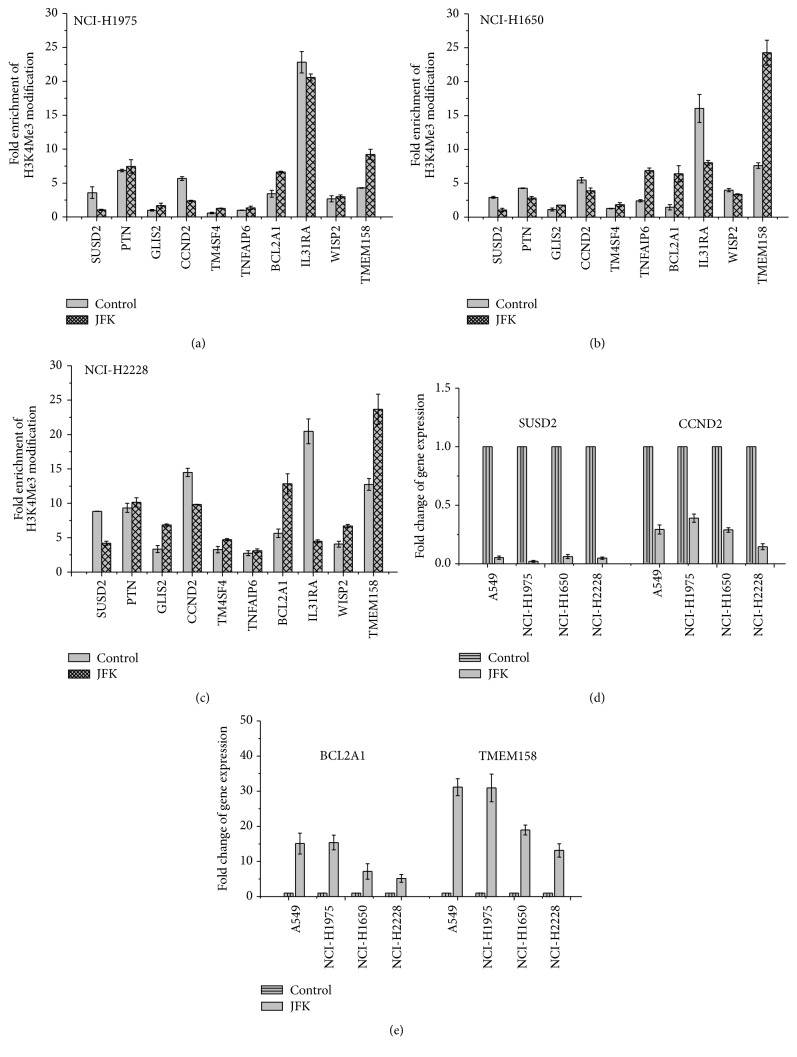
Analysis of JFK-induced H3K4Me3 pattern and mRNA levels alteration in human lung cancer cell lines. Cells were treated with JFK for 48 h and fold change of H3K4Me3 modification was detected by ChIP-qPCR (*n* = 3). (a) NCI-H1975 cell line. (b) NCI-H1650 cell line. (c) NCI-H2228 cell line. Cells were treated with JFK for 48 h and the mRNA levels of* SUSD2*,* CCND2*,* BCL2A1,* and* TMEM158* were detected by RT-qPCR (*n* = 3). (d) Fold change of* SUSD2* and* CCND2* expression. (e) Fold change of* BCL2A1* and* TMEM158* expression.

The above results suggest that JFK-induced A549 cell apoptosis could be due to changes in the H3K4Me3 modification levels around the TSSs of some important genes. Therefore, we performed ChIP-qPCR to examine the H3K4Me3 modification status of genes mentioned in the previous section in NCI-H1975, NCI-H1650, and NCI-H2228 cells treated with JFK for 48 h. We found that the levels of H3K4Me3 modification at* SUSD2* and* CCND2* are significantly decreased and those at* BCL2A1* and* TMEM158* are markedly increased in each of these cell lines (Figures 6(a)–6(c)). Furthermore, an examination of the gene expression levels demonstrated that the levels of H3K4Me3 modification are indeed positively correlated with the levels of gene expression (Figures 6(d) and 6(e)).

## 4. Discussion

The traditional Chinese medicine formula, JFK, had been used in clinical practice for treating human lung cancer patients for more than ten years [17]. Although the effectiveness of JFK has been well recognized by patients and physicians, the cellular and molecular mechanism remains unclear. Previous study demonstrated that JFK could modulate the expression of genes that are involved in the cell cycle and apoptosis [18]. Therefore, the objective of the present study was to understand the relationship between changes at the cellular level and changes in the degree of H3K4Me3 modification and attempt to identify interesting gene targets that could play an important role in JFK-induced lung cancer cell apoptosis.

Previous studies have suggested that herbal formulas that suppress tumor growth and promote tumor cell apoptosis can be attributed to an inhibition of cell proliferation and activation of cell apoptosis [16, 26]. Due to the complicated composition of herbal formulas, serum pharmacology of Traditional Chinese Medicine has been developed to understand the mechanism of TCM [27, 28]. In this study, we examined the effects of JFK serums on cell growth and the cell nucleus in A549 cells. These results suggest that JFK lowered cell viability, inhibited cell growth, and altered cell nucleus morphology. Previous work has also demonstrated that tumor cells can be induced to death or apoptosis when they are exposed to formula extracts for fixed time [29, 30]. Our results suggest that many A549 cells are induced to death or apoptosis after exposure to JFK for 48 h. Since the levels of H3K4Me3 modification at promoter regions have been linked to disease development, therapy, and prognosis, we performed ChIP-qPCR assays to examine the levels of H3K4Me3 modification at two oncogenes (*MYC* and* EGFR*) in control- and JFK-treated A549 cells, respectively. Previous studies found that there is a significant enrichment of H3K4Me3 modification in the TSS regions of* MYC* and* EGFR* genes in many tumor cells [31, 32]. Our results not only verified these earlier results in A549 cells, but also suggested that JFK can significantly reduce the levels of H3K4Me3 modification at these genes.

H3K4Me3 is an active histone modification, which can positively regulate gene expression [3, 6]. Previous studies suggested that the levels of H3K4Me3 modification of many genes can be altered after exposure to exogenous chemicals or metals [33, 34]. Previous study revealed significant changes in gene expression and protein expression after JFK treatment [18]. However, the underlying epigenetic mechanism remained unclear. Here, we performed genome-wide mapping of H3K4Me3 deposition in the promoter regions within 2000 bp flanking the TSSs by ChIP-seq to enable characterization of the levels of H3K4Me3 modification at nearly all genes with or without JFK treatment in A549 cells. We found that the genes with altered H3K4Me3 modification induced by JFK are enriched in multiple signal pathways. Tumor proliferation and apoptosis related signal pathways are known as important targets for many antitumor drugs [30]. Here, Gene Ontology analysis indicated that JFK-induced A549 cell proliferation inhibition and apoptosis promotion could be due to the alteration of levels of H3K4Me3 modification on genes that belong to multiple tumor-related pathways including pathways in cancer, basal cell carcinoma, apoptosis, regulation of transcription (DNA-templated), and regulation of peptidase activity.

Previous study has suggested that* SUSD2* interacts with galectin-1 (Gal-1), a 14 kDa secreted protein that is synthesized by carcinoma cells and promotes tumor immune evasion, angiogenesis, and metastasis [35].* CCND2* is a cyclin whose function is to regulate the subunit of* CDK4* or* CDK6* and whose activity is required for cell cycle G1/S transition [36]. We found that the JFK-induced cell proliferation arrest could be owing to a reduction in the levels of H3K4Me3 modification at* SUSD2* and* CCND2* in A549 cells.* BCL2A1* is well-known antiapoptosis protein that converts into a proapoptotic protein when bound to NuBCP-9 [37].* TMEM158* is a transmembrane protein that is upregulated in response to activation of the Ras pathway and has been proposed to be a tumor-suppressor gene and a target gene in the mutator pathway [38]. The present study suggests that the increase in the H3K4Me3 modification levels following JFK treatment at* BCL2A1* and* TMEM158* could explain JFK-induced A549 cell apoptosis. We note that, for other important genes, the significant changes in H3K4Me3 modification observed in A549 cells after exposure to JFK for 48 h are not also found in NCI-H1975, NCI-H1650, and NCI-H2228 cell lines.

Taken together, this work presents the first report of JFK inducing proliferation inhibition and the promotion of apoptosis in A549 cells through a possible epigenetic mechanism and shows that JFK significantly altered the H3K4Me3 histone modification levels at many genes that play critical roles in tumor survival related pathways. In particular,* SUSD2, CCND2, BCL2A1,* and* TMEM158* could be potential targets of JFK in human lung cancers. Further studies are needed to assess the mechanism by which the changes in the levels of H3K4Me3 modification at these genes lead to changes in gene or protein expression and eventually understand the utility of epigenetically altered genes as drug targets in the treatment of human lung cancers.

## Supplementary Material

Table S1: H3K4Me3 profiling reveals JFK-induced alterations of H3K4Me3 modification in A549 cells. Comparing JFK-treated versus control HeK4Me3 ChIP data sets, top 2000 altered genes with down-regulated and up-regulated respectively, are listed.

## Figures and Tables

**Table 1:** Primer sequences for ChIP-qPCR and RT-qPCR.

Type	Gene	GenBank	Forward (5′-3′)	Reverse (5′-3′)
ChIP-qPCR	*MYC*	[NM_002467]	CATTCCTGCGCTATTGACAC	AAAAACCATTCCCGTTTTCC
	*EGFR*	[NM_005228]	AGGGAAGCTGAGGAAGGAAC	CGGCTTCAGTTTGAGACCTG
	*SUSD2*	[NM_019601]	AGCCTCTGTCCATCTGTCAG	TTCTTCCCATCACCCCTAGC
	*PTN*	[NM_002825]	GAGCCCTCCGAGAAATCGTA	CAACAAAGGCAGACTGAGCG
	*GLIS2*	[NM_032575]	GGTGCCAAAGGATGTTGCTT	CACAAGGGCAGGATGGTTTG
	*CCND2*	[NM_0201759]	CGCGTTCCCTAGTTTCTGTC	GAAACTTGAAGGGGTGAGCG
	*TM4SF4*	[NM_004617]	GAGGCAGTGAGGAGCTTTTG	AAAGCAAGGGGAATGAGGGT
	*BCL2A1*	[NM_004049]	TATCCACATCCGGGGCAATT	ACGGCATCATTAACTGGGGA
	*IL31RA*	[NM_139017]	GCAGAGTGTCAGCTTGTTCC	TTGCGGACATTCACAGACAC
	*WISP2*	[NM_003881]	CTTGGGTCAGCTCTGCAAAG	CTGCTGTAGTTGTGAAGCCC
	*TNFAIP6*	[NM_007115]	TGCTTCATGGACTCTGAGCT	TTTCTGTACTGCAAGCTGGC
	*TMEM158*	[NM_015444]	TTTGGAAAGGCTGGGAAACG	GGGAGAGGGCACGATTAGAA

RT-qPCR	*SUSD2*	[NM_019601]	CATGAAGCCAGCCCTCCT	GTCGGGTGGCAGGAACAT
	*CCND2*	[NM_0201759]	CTCCTACTTCAAGTGCGTGC	TCGCACTTCTGTTCCTCACA
	*BCL2A1*	[NM_004049]	AGGTGTGTGATTGTGCCATT	AATTGCCCCGGATGTGGATA
	*TMEM158*	[NM_015444]	GCCTAGACTTCAGCCTGGAG	ACCAGGGTCATGAAGCAGG

**Table 2:** The statistics of ChIP-seq data sets.

	Total reads	Unique mapping reads	Mapping rate	Sequencing quality (Q30)
Control input	36251128	31414275	86%	97.49
Control	26353782	20332230	77%	97.13
JFK input	17592377	10755542	61%	96.1
JFK	16435835	12904340	79%	97.22

**Table 3:** GO analysis of top 2000 downregulated genes and upregulated genes, respectively.

Category	Item	Count	*P* value
Downregulated			
GO:0006811	Ion transport	112	2.02*E* − 05
GO:0016055	Wnt receptor signaling pathway	29	8.47*E* − 05
GO:0051347	Positive regulation of transferase activity	43	1.66*E* − 04
GO:0006350	Transcription	255	1.67*E* − 04
GO:0007242	Intracellular signaling cascade	162	2.22*E* − 04
GO:0051338	Regulation of transferase activity	59	2.88*E* − 04
GO:0033674	Positive regulation of kinase activity	41	2.95*E* − 04
GO:0043549	Regulation of kinase activity	57	3.12*E* − 04
GO:0045859	Regulation of protein kinase activity	55	4.21*E* − 04
GO:0045860	Positive regulation of protein kinase activity	39	5.70*E* − 04

Upregulated			
GO:0012501	Programmed cell death	65	1.94*E* − 06
GO:0006917	Induction of apoptosis	39	3.86*E* − 06
GO:0050870	Positive regulation of T-cell activation	14	4.85*E* − 05
GO:0006915	Apoptosis	65	4.97*E* − 04
GO:0006631	Fatty acid metabolic process	27	5.34*E* − 04
GO:0012502	Induction of programmed cell death	39	5.74*E* − 04
GO:0031348	Negative regulation of defense response	9	8.36*E* − 04
GO:0052547	Regulation of peptidase activity	15	1.15*E* − 03
GO:0006793	Phosphorus metabolic process	97	1.38*E* − 03
GO:0006796	Phosphate metabolic process	97	1.72*E* − 03

## Abbreviations

JFK: Jinfukang formula
TCM: Traditional Chinese medicine
ChIP-seq: Chromatin immunoprecipitation followed by high-throughput sequencing
TSSs: Transcription start sites
CCK8: Cell Counting Kit 8
DAPI: 4,6-Diamidino-2-phenylindole-dihydrochloride
PI: Propidium iodide
DAVID: Database for Annotation, Visualization, and Integrated Discovery
GO: Gene Ontology
KEGG: Kyoto Encyclopedia of Genes and Genomes.
